# Fiberoptic Endoscopic Evaluation of Swallowing (FEES) in Head and Neck Cancer Patients with Late Radiation-Associated Dysphagia: Swallowing Safety, Efficacy, and Dysphagia Phenotype

**DOI:** 10.3390/curroncol32040233

**Published:** 2025-04-16

**Authors:** Marco Gitto, Francesco Mozzanica, Vincenzo Porpiglia, Luca Morelli, Aurora Ninfa, Alessandro Selvagio, Sara Rocca, Nicole Pizzorni, Antonio Schindler

**Affiliations:** 1 Department of Phoniatrics and Logopedics, Buzzi Children’s Hospital, 20154 Milan, Italy; vincenzo.porpiglia@unimi.it (V.P.); luca.morelli@unimi.it (L.M.); aurora.ninfa@unimi.it (A.N.); selvagio.alessandro@asst-fbf-sacco.it (A.S.); sara.rocca@unimi.it (S.R.); nicole.pizzorni@unimi.it (N.P.); antonio.schindler@unimi.it (A.S.); 2 Department of Biomedical and Clinical Sciences, Università degli Studi di Milano, 20157 Milan, Italy; 3 Department of Clinical Sciences and Community Health, Università degli Studi di Milano, 20122 Milan, Italy; francesco.mozzanica@unimi.it; 4 Department of Otorhinolaryngology, IRCCS Multimedica, 20123 Milan, Italy

**Keywords:** fiberoptic endoscopic evaluation of swallowing, head and neck cancer, radiation-associated dysphagia, swallowing safety, swallowing efficacy, dysphagia phenotypes

## Abstract

Late radiation-associated dysphagia (late-RAD) remains a challenge in head and neck cancer (HNC) survivorship, despite advancements in treatment methods. Although Fiberoptic Endoscopic Evaluation of Swallowing (FEES) stands as the preferred diagnostic approach for oropharyngeal dysphagia assessment in the HNC population, current studies lack a FEES-derived swallowing parameter characterization and phenotypic classification within this specific cohort. This study sought to employ FEES-based assessment to characterize swallowing safety and efficacy profiles, identify distinct phenotypes in HNC patients suffering from late-RAD, and examine potential correlations between safety and efficacy parameters. A retrospective analysis included twenty-four post-radiotherapy HNC patients evaluated using standardized FEES protocols across three bolus consistencies (liquid, semisolid, and solid). Swallowing safety was quantified using the Penetration–Aspiration Scale (PAS), while efficacy was measured via the Yale Pharyngeal Residue Severity Rating Scale (YPRSRS). Additionally, six distinct dysphagia phenotypes were characterized within the cohort. Propulsion deficit was the predominant phenotype (92%), followed by delayed pharyngeal phase (37.5%) and protective deficit (25%), with 46% of patients exhibiting multiple phenotypes. Unsafe swallowing occurred most frequently with liquid consistency (62.5%), while residue was most prevalent with semisolid (82.6% valleculae, 52.2% pyriform sinuses) and solid consistencies (92.3% valleculae, 53.8% pyriform sinuses). Significant correlations were found between penetration–aspiration and pharyngeal residue scores across consistencies (*p* < 0.05). FEES examination revealed distinct phenotypes in late radiation-associated dysphagia, with a predominance of propulsion deficit and significant interdependence between safety and efficacy parameters.

## 1. Introduction

Treatment approaches for head and neck cancer (HNC) have advanced considerably in recent decades, with radiotherapy demonstrating equivalent survival outcomes to surgery as primary treatment or being used alongside surgery and chemotherapy [1,2]. However, long-term studies have revealed complex mortality patterns: approximately 40% of patients die within 5 years, with 25% of deaths attributed to non-cancer causes and 15–20% to unknown reasons [2,3]. While advanced radiation strategies have improved cancer-specific disease-free survival, ten-year follow-up data highlight the emergence of challenges in survivorship care, with late radiation-associated dysphagia (late-RAD) remaining a primary issue [1,2,3,4].

Fiberoptic Endoscopic Evaluation of Swallowing (FEES) serves as one the main tools for dysphagia assessment, with otolaryngologists and speech and language pathologists applying FEES across various clinical scenarios [5,6]. The method proves particularly valuable across different clinical settings, including acute care and rehabilitation facilities [5]. However, the existing evidence base for radiation-associated dysphagia relies predominantly on videofluoroscopic studies (98.2% utilization), creating a discrepancy between research findings and daily practice [6,7]. While Videofluoroscopic Swallow Study (VFSS) provides detailed visualization of the pharyngeal and esophageal phases through its radiographic approach, FEES presents unique benefits in assessing laryngeal sensitivity and secretion management—two key elements in post-radiation dysphagia evaluation and silent aspiration risk assessment. Notably, FEES is the preferred method for assessing oropharyngeal dysphagia in HNC patients, with 82% of speech–language therapists in the United Kingdom employing FEES for this purpose [2,6]. This gap poses significant challenges given the increasing prevalence of swallowing disorders after radiation and the need for management strategies [2].

Swallowing assessment examines two principal domains: safety and efficacy [8,9]. Safety reflects the degree of airway protection during deglutition, measured through penetration and aspiration events. This parameter bears direct clinical relevance through its association with pneumonia risk and mortality rates [10]. Efficacy represents the completeness of bolus transport through the pharynx, measured by residue quantity and location [11]. Efficacy deficits pose significant risk for malnutrition and dehydration, negatively impacting overall health outcomes and quality of life in cancer survivors. Safety and efficacy parameters often demonstrate interdependence, with accumulated residue increasing post-swallow aspiration risk [10,12].

Three knowledge gaps limit post-radiation swallowing dysfunction research: incomplete FEES-based swallowing parameter data, undefined relationships between swallowing safety and efficacy, and lack of phenotypic classification [13,14]. A phenotype represents observable physiological and functional characteristics that manifest as distinct patterns of swallowing dysfunction. These patterns can be identified through fiberoptic endoscopic or videofluoroscopic examination in two primary ways: through post-swallow accumulation patterns (such as the “residue phenotype” showing accumulation in valleculae and pyriform sinuses) and through airway protection deficits (the “penetration-aspiration phenotype”). More specifically, six distinct phenotypes were identified: protective deficit affecting laryngeal elevation and glottic closure, posterior oral incontinence with compromised bolus containment, delayed pharyngeal phase, oropharyngeal dyspraxia, propulsion deficit, and resistive issue. These phenotypes may present either in isolation or, more commonly, as combined patterns affecting multiple components of the swallowing mechanism simultaneously [15,16,17]. Similar frameworks have improved neurogenic dysphagia evaluation, particularly in stroke patients [15,16,18] and children with spinal muscular atrophy type 1 [19], but radiation-associated cases lack such standardized categories. Establishing a structured phenotype-based system that accounts for anatomical–radiation interactions could facilitate targeted rehabilitation strategies with measurable benchmarks and guide the selection of compensatory techniques.

This study examined two objectives: (a) characterization of swallowing safety and efficacy, dysphagia phenotypes through FEES-based assessment in HNC patients with late-RAD, and (b) analysis of correlations between swallowing safety and efficacy parameters [14,17,19,20]. Based on previous VFSS studies [21,22], we hypothesized that a high percentage of penetration/aspiration and pharyngeal residue will be observed in this population. As this represents the first study to analyze dysphagia phenotypes using FEES in radiation-associated dysphagia, it is difficult to predict with certainty which phenotype will predominate, although propulsion deficit is expected to be a common mechanism given the documented effects of radiation on pharyngeal musculature [21,23]. Regarding the relationship between swallowing safety and efficacy, we hypothesized that an association exists between penetration/aspiration and pharyngeal residue, though this specific correlation has not been previously analyzed in the post-radiation HNC population. Clinical applications of this study may contribute to the development of more targeted assessment protocols for late radiation-associated dysphagia and provide initial evidence for phenotype-based clinical decision making in FEES evaluations. These findings could enhance our understanding of swallowing dysfunction patterns in this specific population, supporting more informed diagnostic approaches.

## 2. Materials and Methods

### 2.1. Participants

Patients were selected from all cases who underwent radiotherapy for HNC and were referred to the Phoniatric Department for dysphagia evaluation between January 2021 and December 2023. All the enrolled patients were treated with radiotherapy (+/− chemotherapy) with curative intent for HNC. Participants were eligible for study inclusion if they satisfied the following parameters: adult status (≥18 years of age); oral intake encompassing multiple food consistencies; adequate dentition (either complete natural dentition or partial edentulism rehabilitated with appropriate prosthetic restoration) [24]; no medical history of gastroenterological, respiratory, rheumatologic, metabolic, hematologic disorders. Exclusion criteria included a history of distant metastases, previous surgery of the HN, second primary or recurrent/residual disease, and any comorbidity that could impact swallowing function (e.g., stroke).

Oral intake status was assessed using the Functional Oral Intake Scale (FOIS) [25,26,27], a validated 7-point ordinal metric that quantifies feeding restrictions from level 1 (complete absence of oral intake) to level 7 (unrestricted oral alimentation). Administration of the FOIS was completed immediately preceding the fiberoptic endoscopic evaluation procedure. According to the results of the FEES examination and diet recommendation, the FOIS score was rated again. The FOIS scale was selected because it has been validated in Italian [27] and it is widely used in dysphagic population. Information regarding the body mass index (BMI) was also collected.

### 2.2. FEES Examination

FEES examinations were conducted by senior phoniatricians employing a XION EF-N flexible endoscope (3.4 mm diameter, 320 mm length; XION GmbH, Berlin, Germany) integrated with an EndoSTROB E camera system (XION GmbH, Berlin, Germany). Video recordings underwent processing via Daisy Viewer 2.0 software (INVENTIS srl, Padua, Italy) and were archived in anonymized .AVI format.

Participants were positioned in reclined chairs (75–90° incline) with supported upper extremities and neutral cranial alignment to facilitate optimal visualization. To maintain intact pharyngo-laryngeal sensitivity, no topical anesthetics were administered [28]. The endoscope was introduced via the most patent nasal passage and positioned sub-uvularly to ensure a comprehensive visualization of laryngeal structures, glossoepiglottic valleculae, and pyriform sinuses [5,29,30].

Swallowing assessment protocol incorporated three bolus consistencies: liquid trials utilizing room-temperature blue-dyed water (<50 mPa·s at both 50 s^−1^ and 300 s^−1^; IDDSI Level 0) [30]; semisolid trials employing room-temperature Crème Line vanilla pudding (Nutrisens Medical s.a.s.e modified because it should be s.a.s (società in accomandita semplice (S.a.s.), Francheville, France) with rheological properties of 2583.3 ± 10.41 mPa·s at 50 s^−1^ and 697.87 ± 7.84 mPa·s at 300 s^−1^ (IDDSI Level 4); solid trials incorporating fractional portions (one-quarter and one-half) of an 8 g dry biscuit (4 g per trial; IDDSI Level 7).

FEES examinations were rated independently by three operators using the video files. All of them were phoniatricians with at least 4 years of experience in FEES examinations. To ensure assessment objectivity, phoniatricians evaluated anonymized video recordings independently and without access to participants’ clinical data. Two autonomous specialists rated each recording utilizing validated ordinal scales for swallowing safety and efficacy parameters, with subsequent analysis of inter-rater reliability. When rating discrepancies exceeded one level on any FEES assessment scale, adjudication by a third expert phoniatrician determined the final ratings for both parameters [28].

The FEES protocol facilitated evaluation of multiple dysphagia parameters, including phenotypic classification according to Desuter’s videoendoscopic taxonomy [31]. Six distinct phenotypes were systematically assessed: (1) protective deficit, encompassing impairments in laryngeal elevation, glottic closure, and tongue propulsion mechanisms; (2) posterior oral incontinence, characterized by the inability to maintain bolus containment within the oral cavity upon request; (3) delayed pharyngeal phase, defined as temporal latency in at least one component (arytenoid approximation/glottic closure, laryngeal elevation, or tongue base propulsion), resulting in pre-swallow bolus migration into piriform sinuses beyond the glossopharyngeal ligaments; (4) oropharyngeal dyspraxia, manifested as absence of pharyngeal swallowing with consequent oral bolus retention or cyclical aborted tongue base retraction movements; (5) propulsion deficit, evidenced by vallecular and/or piriform sinus residue associated with inadequate tongue base retraction, pharyngeal peristalsis, and/or laryngeal elevation; (6) resistive issue, identified by presence of retrocricoid residue. All the different scenarios that were found within the same patient were included in the analysis.

• Safety impairment (Penetration/aspiration) was quantified utilizing the Penetration–Aspiration Scale (PAS) [32], an 8-point ordinal metric ranging from 1 (complete airway protection) to 8 (subglottic aspiration without ejection attempt). Penetration was operationally defined as the supraglottic entry of bolus material (PAS 2–5), while aspiration was characterized by infraglottic bolus passage (PAS ≥ 6). Consistent with our previous investigations [14,17,19], swallowing events were classified as functionally unsafe when material breached the laryngeal vestibule (PAS ≥ 3). For statistical analyses, the most severe PAS score observed per consistency for each participant was utilized.
• Efficacy impairment (pharyngeal residue) was assessed through quantification of pharyngeal residue utilizing the Yale Pharyngeal Residue Severity Rating Scale (YPRSRS), with separate evaluations conducted for the vallecular and pyriform sinus regions [33,34]. Residue was assessed at the end of the static swallow phase. This standardized timing was maintained across all participants and consistencies to ensure consistent evaluation of initial swallowing efficiency [33,34]. In particular, a YPRSRS score ≥ 3 (mild residue) was considered suggestive of inefficient swallowing. The worst YPRSRS score for each consistency and for each subject was considered for statistical analyses.

### 2.3. Statistical Analysis

The results were reported as median (interquartile ranges). Distribution normality of FEES parameters across the patient cohort was assessed utilizing the Kolmogorov–Smirnov test. Given that results revealed non-Gaussian distribution patterns, subsequent analyses employed non-parametric statistical methodologies. Inter-rater reliability between the two SLTs’ FEES scoring was evaluated through computation of weighted kappa statistics with quadratic weighting [35]. The resultant kappa coefficients were categorized according to established criteria, with values ≤ 0.20 indicating poor agreement, 0.21–0.40 signifying fair agreement, 0.41–0.60 representing moderate agreement, 0.61–0.80 denoting good agreement, and 0.81–1.00 reflecting very good agreement [36]. The Spearman test was used to evaluate the correlation among PAS and YPRSRS scores obtained in the cohort of patients. Correlations were classified as mild for values between 0.30 and 0.50, moderate for values between 0.50 and 0.70, and strong for values greater than 0.70 [37].

## 3. Results

### 3.1. Sample

A total of 24 patients who underwent radiotherapy for HNC were included in the final analysis. Of these, 8 (33.3%) received exclusive RT, while 16 (66.7%) were treated with concurrent C/RT. The demographic characteristics of the enrolled patients are reported in Table 1.

**Figure 1:**
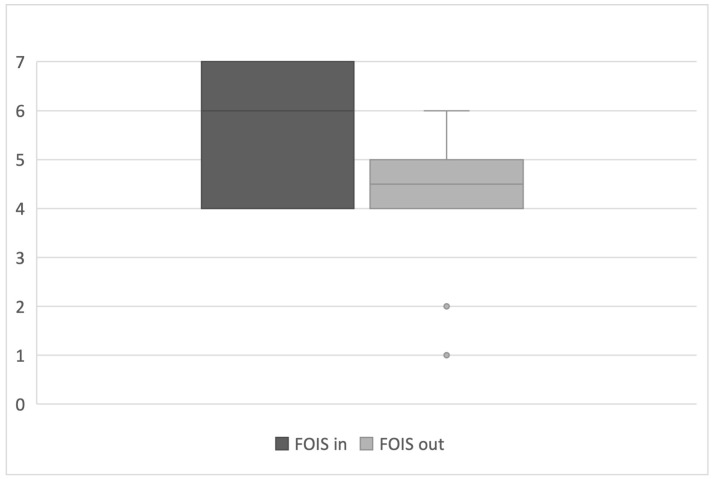
FOIS score before (FOIS in) and after (FOIS out) the Fiberendoscopic Evaluation of Swallowing Evaluation.

The median FOIS score before and after the FEES examination was 5.5 (interquartile range 4–7) and 4.5 (interquartile range 4–5), respectively (Figure 1).

The full FEES protocol was performed on 12 patients. Solid consistency was not tested in 11 patients (30.6%), while semisolid was not tested in 1 (2.8%) because of a significant risk of choking. Procedural duration of FEES examination consistently remained under 15 min. Concordance between raters for FEES assessments demonstrated robust reliability indices. Specifically, inter-examiner agreement across all bolus consistencies yielded very good reliability coefficients for the PAS (*k* > 0.88) and for the YPRSRS in both the vallecula (*k* > 0.83) and pyriform sinus (*k* > 0.87).

### 3.2. Dysphagia Phenotypes

**Figure 2:**
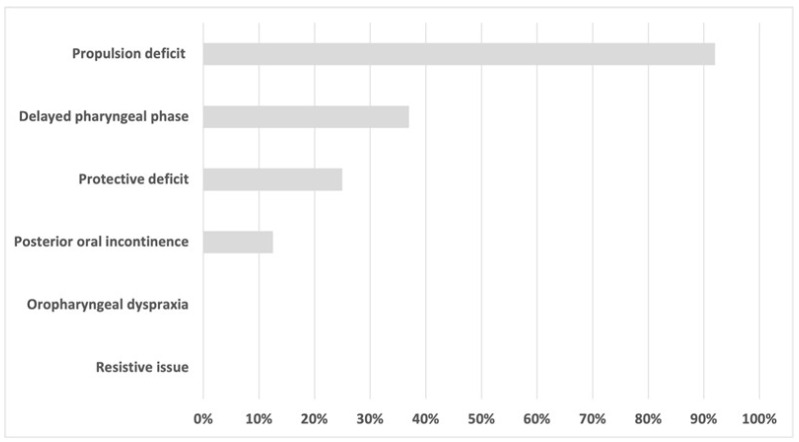
Dysphagia phenotypes in the total cohort. The results are reported as percentages.

The dysphagia phenotypes in the cohort of patients are reported in Figure 2.

The propulsion deficit represented the most common phenotype (92%, 22 out of 24 patients), followed by delayed pharyngeal phase (37.5%, 9 out of 24 patients) and protective deficit (25%, 6 out of 24 patients). Only one patient demonstrated normal deglutition. Twelve (50%) patients showed only one isolated phenotype, four (17%) patients showed two combined phenotypes, and seven (29%) patients showed three combined phenotypes.

### 3.3. Swallowing Safety

**Figure 3:**
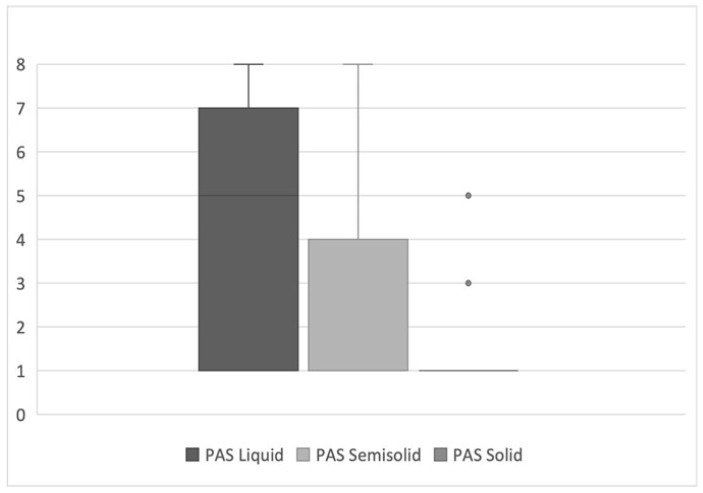
Distribution of the Penetration–Aspiration Scale (PAS) scores in the cohort of patients.

The distribution of the PAS scores is reported in Figure 3.

PAS assessment revealed notable patterns across different consistencies. Overall, penetration or aspiration occurred most frequently with liquids (69.4% of patients), followed by semisolids (34.3%), with solids showing the lowest aspiration rate (8.3%). Silent aspiration (PAS 8) was observed most frequently with liquid consistency (5.6% of patients), followed by semisolid (2.9%), with no cases observed with solid consistency.

### 3.4. Swallowing Efficacy

**Figure 4:**
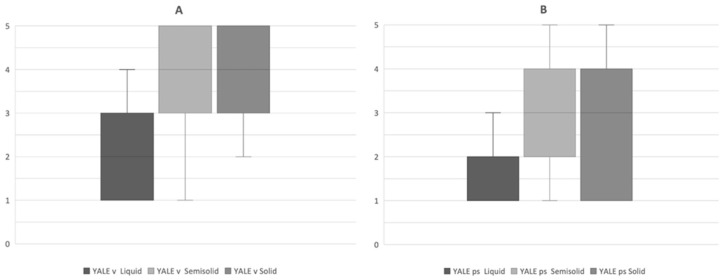
(**A**) Distribution of the Yale (**B**) Pharyngeal Residue Rating Scale (YALE) valleculae (v) and pyriform sinuses (ps) scores obtained from the cohort of patients.

The distribution of YPRSRS scores is reported in Figure 4.

Analysis of swallowing efficacy revealed consistency-dependent patterns of post-deglutitive residue. During liquid bolus trials, minimal or greater vallecular and pyriform sinus residue was observed in 7/24 (29.2%) and 5/24 (20.9%) participants, respectively. Following semisolid bolus administration, the prevalence of minimal or greater residue increased substantially to 19/23 (82.6%) in the valleculae and 12/23 (52.2%) in the pyriform sinuses. The highest residue burden occurred during solid consistency challenges, where 12/13 (92.3%) of participants exhibited minimal or greater vallecular residue and 7/13 (53.8%) demonstrated pyriform sinus residue.

### 3.5. Association Analysis

The correlations among the PAS and YPRSRS scores obtained in the cohort of patients are reported in Table 2.

Significant correlations were demonstrated in Spearman test between PAS scores for liquid and semisolid on one side and the YPRSRS valleculae and pyriform sinuses for liquid and semisolid on the other, with correlation coefficients ranging from moderate (*r* = 0.438) to strong (*r* = 0.701). PAS score for solid was significantly correlated only with the YPRSRS pyriform sinus scores for semisolid and solid (*r* = 0.665 and *r* = 0.592, respectively).

## 4. Discussion

The dysphagia characteristics in patients with late-RAD have been evaluated using FEES examination. For the first time, distinct swallowing phenotypes have been identified through FEES assessment in this population, along with characterization of swallowing safety and efficacy across different consistencies.

### 4.1. Dysphagia Phenotypes

Propulsion deficit emerged as the predominant feature, followed by delayed pharyngeal phase and protective deficit. The high prevalence of combined phenotypes (46% of patients showing multiple patterns) suggests complex pathophysiological mechanisms underlying radiation-associated dysphagia. While no previous studies have analyzed dysphagia phenotypes using FEES in this population, our findings align with VFSS studies examining swallowing dysfunction patterns on radiation-associated dysphagia and late-RAD. In particular, Dumbak et al. [38] documented that pharyngeal phase scores increased in the 3rd month after treatment and remained high in the 6th month, with the “pharyngeal stripping wave” component showing significant deterioration related to radiation exposure of superior pharyngeal constrictors. Additionally, Hutcheson et al. [21] showed that late radiation-associated dysphagia is characterized by progressive deterioration of pharyngeal function over time, with higher risk of aspiration in long-term survivors. Similarly, Gharzai et al. [39] documented that at least 15.1% of oropharyngeal cancer patients experience moderate dysphagia more than 2.5 years after treatment, with 7.5% showing patterns of late progressive dysphagia. Through FEES evaluation, our study provided visualization of the resulting pharyngeal residue and airway protection deficits in late post-treatment dysphagia, offering complementary information to previous VFSS findings that focused on pharyngeal contraction and bolus transport mechanics.

### 4.2. Swallowing Safety

Swallowing safety analysis in our cohort revealed unsafe swallowing occur most frequently with liquid consistency, followed by semisolid and solid consistencies. The aspiration rate in our study was higher than that reported by Dumbak et al. [38], who found aspiration in 18% of patients at 6 months post-radiotherapy. When compared with the existing literature on late radiation-associated dysphagia, our aspiration rates align with some of the findings. Hutcheson et al. [40] reported that up to 85% of long-term survivors with late-RAD developed pneumonia as a consequence of intractable aspiration, and two-thirds of late-RAD cases ultimately required lifelong gastrostomy despite rehabilitation efforts. Similarly, Awan et al. [22] found that 83% of late-RAD cases (10 of 12 patients) presented lower cranial neuropathies that contributed to their dysphagia, manifesting as “a highly inefficient swallow with substantial pharyngeal residue and a tendency for silent aspiration”. In their study, only one late-RAD case had stricture co-existing with physiological pharyngeal dysphagia, similar to the primary propulsion deficit phenotype we observed. However, interpretation of our solid consistency data requires caution, as this consistency could not be tested in 11 patients due to safety concerns. This methodological limitation suggests our solid consistency data might underestimate the true prevalence of safety issues in this population.

### 4.3. Swallowing Efficacy

The efficacy analysis demonstrated substantial residue across consistencies, with distinctive accumulation profiles in the valleculae versus pyriform sinuses. With liquid consistency, residue was found in 29.2% and 20.9% of patients in the valleculae and pyriform sinuses, respectively. Residue was particularly prevalent with semisolid (82.6% valleculae, 52.2% pyriform sinus and solid consistencies (92.3% valleculae, 53.8% pyriform sinuses). In Liou at et al.’s [41] paper, efficacy was assessed using the Bolus Residue Scale (BRS) [42]. Their data showed no significant differences in BRS scores among different tumor sites, with median BRS scores of 3 across all consistencies. This contrasts with our findings where residue patterns showed marked differences across consistencies, particularly affecting semisolid and solid boluses. The differences might be attributed to our use of the YPRSRS, which offers a distinct analysis of the location of the residues between the valleculae and the pyriform sinuses, versus their use of the BRS scoring system. The high prevalence of residue with thicker consistencies in our study suggests significant impairment in pharyngeal clearance mechanisms, likely reflecting radiation-induced changes in muscle function and tissue properties. This interpretation is supported by previous research documenting radiation’s impact on pharyngeal muscle function [43,44,45].

### 4.4. Association Between Swallowing Safety and Efficacy Parameters

The correlation analysis between swallowing safety and efficacy parameters revealed significant relationships across consistencies. Significant correlations were found between PAS scores for liquid and semisolid consistencies and the YPRSRS valleculae and pyriform sinus scores for the same consistencies (*p* < 0.05). PAS scores for solid consistency showed significant correlation only with YPRSRS pyriform sinus for semisolid and solid. These findings indicate that pharyngeal residue accumulation and penetration–aspiration are interdependent phenomena, suggesting that residue may predispose patients to unsafe swallowing. These correlations are consistent with, though stronger than, those reported by Guijo et al. [18] in post-stroke patients, who found moderate correlations between YPRSRS in vallecula (r = 0.43), pyriform sinus (r = 0.54), and the combined sites (r = 0.57) with PAS scores. Similarly, Shapira-Galitz et al. [7] demonstrated significant correlations between pharyngeal residue severity and PAS across all consistencies tested (liquid, purée, solid) in a heterogeneous dysphagia population, with purée showing the strongest correlation (PCC = 0.631). The correlation strength in our HNC cohort, particularly for pyriform sinus residue, suggests that radiation-associated pharyngeal muscle fibrosis could predispose to an aspiration risk that differs from stroke-related or heterogeneous dysphagia pathophysiology. This difference likely reflects the presence of tissue damage caused by radiotherapy, affecting both the sensory feedback mechanisms and motor control required for pharyngeal clearance [23].

### 4.5. Study Strength and Limitation

Our findings have implications for assessment and management of radiation-associated dysphagia. The consistency-dependent patterns observed in both safety and efficacy domains suggest that dietary modifications should be guided by comprehensive instrumental assessment rather than generalized recommendations. This is particularly relevant given the observed reduction in FOIS scores post-examination, indicating that objective FEES findings often lead to more conservative dietary management than clinical evaluation of swallowing alone.

The study has methodological strengths in its approach to phenotype classification and standardized FEES protocol implementation. However, several limitations need consideration. The cross-sectional design restricts the evaluation of dysphagia pattern progression over time, and the sample size, though sufficient for primary analyses, may limit the identification of less common phenotypic presentations. A notable methodological limitation is the use of convenience sampling, as patients were enrolled from those referred to the phoniatric unit for dysphagia evaluation, which may introduce selection bias and potentially affect the representativeness of the findings for a broader HNC post-radiation population. Additionally, the varied completion rates across consistencies (50% full protocol completion) should be considered when interpreting consistency-specific findings, as patients with more severe impairments may not have been evaluated with all consistencies, potentially underestimating the true prevalence of dysphagia patterns, particularly for solid consistency. Furthermore, the number of patients in our cohort with hypopharyngeal (*n* = 2, 8.3%) and laryngeal (*n* = 4, 16.7%) tumors was relatively low. This underrepresentation could account for the absence of certain dysphagia phenotypes, such as resistive issues or upper esophageal sphincter fibrosis patterns, which are seen with extensive hypopharyngeal or laryngeal involvement. Consequently, our findings should be interpreted with caution, as the distribution of tumor sites may have influenced the phenotypic presentation. Another limitation of our study is the relatively small sample size, which did not allow us to adequately assess whether distinct tumor sites (e.g., larynx vs. oropharynx) or tumor stages (T1–T2 vs. T3–T4) correlate with specific dysphagia phenotypes.

## 5. Conclusions

This study provides FEES-based characterization of swallowing dysfunction in patients with late-RAD. Our findings revealed distinct phenotypic presentations with propulsion deficit as the predominant feature, often occurring in combination with delayed pharyngeal phase and protective deficit. Swallowing safety was most compromised with liquid consistency, while efficacy was particularly impaired with thicker consistencies. The significant correlations between penetration–aspiration and pharyngeal residue across consistencies demonstrate the interdependence between safety and efficacy parameters in this population. These phenotypes reflect the pathophysiological consequences of radiation therapy on pharyngo-laryngeal structures, which differ from those seen in other etiologies. Future research should focus on longitudinal evaluations of radiation-associated dysphagia phenotypes. Studies investigating relationships between radiation treatment parameters and dysphagia phenotypes would benefit risk prediction. Additionally, studies examining phenotype-targeted intervention outcomes are needed to optimize rehabilitation approaches in this population. While this study provides insights into the phenotypic presentation of late radiation-associated dysphagia, the sample size limited our ability to evaluate differences according to tumor site and stage. Larger, prospective studies are warranted to confirm our findings, clarify the influence of tumor characteristics on dysphagia phenotypes, and guide more targeted rehabilitation approaches.

## Figures and Tables

**Table 1:** Characteristics of the enrolled population. The results are reported as median (interquartile range); frequencies and percentages (in parentheses) are reported for other variables.

Characteristic		N°	(%)
Sex	Male	14	58.3%
	Female	10	41.7%
Age (years)		62 (56.76–68)	
Tumor location	Rhinopharynx	9	37.5%
	Oropharynx	9	37.5%
	Hypopharynx	2	8.3%
	Larynx	4	16.7%
T classification	T1	1	4.2%
	T2	4	16.7%
	T3	13	54.2%
	T4	6	25.0%
N classification	N0	2	8.3%
	N1	2	8.3%
	N2a	5	20.8%
	N2b	4	16.7%
	N3	6	25.0%
Treatment	Radiotherapy	8	33.3%
	Chemo-radiotherapy	16	66.7%
Years after diagnosis		8 (4–13)	

**Table 2:** Correlation among YPRSRS and PAS scores in the cohort of patients. The results of the Spearman test are reported. * = *p* < 0.05; ** = *p* < 0.01.

		YPRSRS Valleculae			YPRSRS Pyriform Sinuses		
		Liquid	Semisolid	Solid	Liquid	Semisolid	Solid
PAS	Liquid	0.677 **	0.701 **	0.249	0.585 **	0.659 **	0.532
	Semisolid	0.594 **	0.438 **	0.040	0.577 **	0.697 **	0.639 *
	Solid	0.198	0.360	0.005	0.204	0.665 **	0.592 *

## Data Availability

Data relating to this study can be made available by contacting the corresponding author.
